# Sugar-containing beverage intake at the age of 1 year and cardiometabolic health at the age of 6 years: the Generation R Study

**DOI:** 10.1186/s12966-015-0278-1

**Published:** 2015-09-17

**Authors:** Elisabeth T. M. Leermakers, Janine F. Felix, Vincent W. V. Jaddoe, Hein Raat, Oscar H. Franco, Jessica C. Kiefte- de Jong

**Affiliations:** Generation R Study Group, University Medical Center Rotterdam, Rotterdam, The Netherlands; Department of Epidemiology, University Medical Center Rotterdam, room Na 2909, Erasmus MC, P.O. Box 2040, 3000 CA Rotterdam, The Netherlands; Department of Pediatrics, University Medical Center Rotterdam, Rotterdam, The Netherlands; Department of Public Health, Erasmus MC, University Medical Center Rotterdam, Rotterdam, The Netherlands; Leiden University College, The Hague, The Netherlands

**Keywords:** Sugar-containing beverages, Cardiometabolic, Children, Epidemiology, Cohort

## Abstract

**Background:**

Consumption of sugar-containing beverages (SCBs) in adults has been associated with an increased risk of metabolic syndrome. Although the effect of SCB on body weight in children is well established, little is known about the cardiometabolic effects in young children. We studied the associations of SCB intake at the age of 1 year with cardiometabolic health at age 6 years.

**Methods:**

This study was performed among 2,045 Dutch children from a population based prospective birth cohort. SCB intake was assessed with a semi-quantitative food frequency questionnaire at the age of 13 months and sex-specific tertiles were created. Children visited the research center at the age of 6 years. We created a continuous cardiometabolic risk factor score including: body fat percentage, blood pressure, insulin, HDL-cholesterol and triglycerides. Age-and sex-specific standard deviation (SD) scores were created for all outcomes. Multivariable linear regression was performed with adjustment for socio-demographic and lifestyle variables of mother and child.

**Results:**

In the total population, we observed an association between higher SCB intake at 13 months of age and a higher cardiometabolic risk factor score at the age of 6 years (0.13SD (95 % CI 0.01; 0.25), highest vs. lowest tertile) After stratification by sex, we found that boys in the highest tertile of SCB intake had a higher cardiometabolic risk factor score (0.18 SD (95 % CI 0.01; 0.34)), as compared to boys in the lowest tertile of SCB intake. There was no significant association in girls. We did not find associations of SCB intake with the individual cardiometabolic risk factors in the total population, or in the stratified analyses.

**Conclusion:**

Higher SCB intake at 1 year of age was associated with a higher cardiometabolic risk factor score at age 6 years in boys, but not in girls. Further research on sex-specific effects of SCBs is needed.

## Background

Both experimental and observational studies have shown evidence of a relation between consumption of sugar-containing beverages (SCBs) and an increase in body weight in both children and adults [1]. Several studies in adults have also shown an association between consumption of SCBs and an increased risk of metabolic syndrome and type 2 diabetes [2]. Part of this association is explained by an increased risk of obesity [1], but it has been suggested that SCBs may also affect cardiometabolic health independent of weight gain [3].

Also in school-age children, harmful effects have been observed of SCBs on cardiometabolic risk factors, such as on blood pressure, blood lipids and glucose intolerance [4–6]. Recently, we reported that SCB intake in toddlers was associated with higher BMI in school-aged girls [7]. However, associations between SCB consumption in infancy and cardiometabolic outcomes at school age have not been reported thus far. While consumption of sugar-sweetened beverages, such as carbonated soft drinks and sports drinks, is low during infancy, sugar-containing beverages like fruit juices and fruit concentrates, are frequently consumed [8]. These beverages might have a more healthy image because they are fruit-derived and contain valuable nutrients, but they also contain high amounts of sugar [9]. Recently, the WHO launched its’ new guidelines on sugar consumption [10]. SCBs play an important role in this guideline, and the recommendation is strongly to limit the intake of sugars in order to improve health. Despite the suggested adverse effects of SCBs on cardiometabolic health, there are no studies that report on SCBs consumption in very young children in relation to cardiometabolic health. Therefore, we aimed to examine the relation between SCB intake at 13 months, with cardiometabolic outcomes at age 6 years, among Dutch children participating in a population-based prospective cohort study.

## Methods

### Study population

**Fig. 1:**
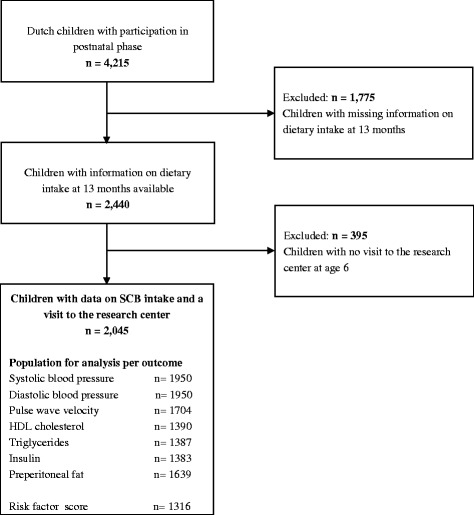
Flow chart of participants included for analysis

This study was performed in children from the Generation R Study, a population-based birth cohort in Rotterdam, the Netherlands, which has previously been described in detail [11]. This study was approved by the Medical Ethical Committee at Erasmus MC, University Medical Center, Rotterdam (MEC 198.782/2001/31). Written informed consent was obtained from all participating mothers. We only included Dutch children for these analyses because the food-frequency questionnaire (FFQ) was designed for dietary assessment of a Dutch population and was validated in Dutch children [12]. Dutch ancestry was defined as having two parents and all grandparents born in the Netherlands. There were 4,215 Dutch children that participated in the postnatal phase of the study. The FFQ was implemented in a later stage and was therefore available in 71 % of the total population. Children without information on SCB intake at 13 months of age (*n* = 1,775) or without a visit to the research center at age 6 years (*n* = 395) were excluded. Because not all children had blood drawn, the population for analysis ranged from 1316 to 1950 children, depending on the outcome of interest (Fig. 1).

### Dietary assessment

At the child’s age of 13 months (median 12.9, IQR 1.2 months), the primary caregiver (which was the mother in 86.2 %, the father in 3.8 %, both in 9.8 %, and someone else in 0.2 %) completed a 211-item semi-quantitative FFQ [12]. The FFQ asked for habitual diet in the last month, thereby covering diet from the age of 12 months onwards. At dietary assessment, only 6.8 % of children received any breastfeeding and all children received complementary feeding. This FFQ was validated against three 24 h-recalls in a representative sample of Dutch children (*n* = 32), which showed an intraclass correlation coefficient of 0.76 for SCB intake [7, 12]. Total SCBs included fruit juices, fruit concentrates, lemonades, softdrinks and sportsdrinks. According to Dutch standard portion sizes [13], one serving was considered to be 150 ml. Energy adjustment was performed using the residual method to account for measurement error [14] and was standardized to the mean energy intake. We created sex-specific tertiles of SCB intake in the 2,045 children with data on SCB intake and a visit to the research center, and the same tertiles were used for all outcomes.

### Cardiometabolic outcomes

At a median age of 5.9 years (95 % range 5.7–6.5 years) children visited our dedicated research facility at the Erasmus Medical Center, Sophia’s Children Hospital. Non-fasting blood samples were drawn by antecubital venipuncture and insulin, C-peptide, total, HDL, and LDL cholesterol, and triglyceride (TG) concentrations were measured with enzymatic methods (using a Cobas 8000 analyser, Roche, Almere, The Netherlands). Quality control samples demonstrated intra-assay and inter-assay coefficients of variation ranging from 0.69 to 1.57 %. Systolic and diastolic blood pressure (SBP and DBP) were measured at the right brachial artery four times with one-minute intervals with the child lying, using the validated automatic sphygmomanometer Datascope Accutor Plus TM (Paramus, NJ, USA) [15]. Mean SBP and DBP were calculated using the last three measurements for all children that had a maximum of one out of these three missing. Mean arterial pressure (MAP) was calculated as MAP = (mean SBP + 2*mean DBP)/3. Carotid-femoral pulse-wave velocity (PWV) was measured using the automatic Complior SP device (Artech Medical, Pantin, France). Preperitoneal fat was measured by abdominal ultrasound and the preperitoneal fat thickness was determined using a linear (L12-5 MHz) transducer which was placed perpendicular to the skin surface on the median upper abdomen. Total body fat was measured by Dual-energy X-ray absorptiometry (DXA) scans (iDXA; General Electric, 2008, Madison, WI, USA) [16]. Percentage body fat (BF%) was calculated as 100 %*[total body fat mass(g)]/[fat mass + lean mass + bone mass(g)]. Body fat percentage was analyzed as part of a separate study focused on body composition [7], and is therefore not presented separately.

In addition to the individual cardiometabolic outcomes, we calculated a continuous score following examples of previously defined metabolic syndrome scores for children [17], including the components: BF%, blood pressure (including DBP and SBP), HDL-cholesterol, triglycerides, and insulin. The cardiometabolic risk factor score was calculated as the sum of age- and sex-specific SD-scores of these five variables, as proposed previously for pediatric populations [17]. The SD scores for HDL-C were multiplied by −1 since a higher HDL-C represents a better cardiometabolic profile. The SD scores for SBP and DBP were multiplied by 0.5 so they each contribute half to the blood pressure component. The cardiometabolic risk factor score was thus calculated as: SDS BF% + 0.5*SDS SBP + 0.5*SDS DBP + SDS TG + (−1*SDS HDL-C) + SDS insulin.

### Covariates

Information on maternal age (continuous), educational level (secondary school or lower vs. higher education), smoking during pregnancy (never smoked or quit when pregnancy was known vs. continued during pregnancy) and folic acid use (never used, started periconceptionally or started in first 10 weeks) was obtained from questionnaires during pregnancy. Maternal weight and height were measured at enrollment and BMI (kg/m^2^) was calculated. Information about breastfeeding duration was derived from a combination of delivery reports and questionnaires at 6 and 12 months of age. TV watching (hours/day) was used as a proxy measure for sedentary behavior and was derived from the questionnaire at age 2 years. Total energy intake (kcal/day) was derived from the FFQ. With the information of the FFQ child diet quality was scored based on the previously created diet quality score for preschool children [18]. In short, this score was developed based on international dietary guidelines as a basis and includes intake of the following ten food groups: high intake of vegetables; fruits; bread and cereals; rice, pasta, potatoes, and legumes; dairy; meat, poultry, eggs and meat substitutes; fish; and fats and oils; and low intake of candy and snacks; and sugar-sweetened beverages [18]. The score ranges from 0 to 10 on a continuous scale, with a higher score representing a healthier diet. For the current study, the score was slightly modified by excluding the SCB component, thus the diet quality score in the current study had a possible range from 0 to 9.

### Statistical methods

Age-and sex-specific SD scores were created for all outcomes based on the total Generation R population with available measurements. Insulin was not normally distributed and was therefore transformed with square root transformation before standardizing. We used linear regression models to assess the association of SCB intake at 13 months and cardiometabolic outcomes. We estimated the SD difference in outcomes for the middle and highest tertile of SCB intake, as compared to the lowest tertile. Trend tests were performed using tertiles as a continuous variable.

Three multivariable models were analyzed: Model A was a crude model that only contained age at dietary assessment and total energy intake (and sex, when analyses were not stratified by sex), model B was a larger multivariable model which additionally included socio-demographic and lifestyle factors and model C was additionally adjusted for child height and weight at age 6 years, since these may be mediators in the association between SCB and cardiometabolic health [7]. Weight was not included in models that included a measure of adiposity as outcome.

Selection of potential confounders was based on the literature, and confounders were included in all models if they changed the effect estimates of our univariate model of SCB intake with the cardiometabolic risk factor score with 5 % or more [19]. Hence, the same multivariable models were used for all outcomes. A cut-off of 5 % change in effect estimate was chosen, because many factors may influence both diet as well as cardiometabolic health, and the study population was seen as large enough for this low cut-off.

With this method, the following covariates were included as confounders; maternal age, BMI, education level, smoking during pregnancy, folic acid supplement use during pregnancy, breastfeeding of the child, and hours of TV watching at age 2. Child Diet Quality Score did not induce a 5 % change but was nonetheless included as covariate to limit residual confounding by overall diet. The following covariates did not induce a 5 % change in the univariate model and were not included in the final models; maternal gestational age at enrollment in the study, child birth weight, age of the biological father, household income, maternal alcohol use during pregnancy, child’s age at introduction of complementary feeding, child’s allergy to cow’s milk, and playing sports at the age of 6 years.

We checked for an interaction with SCB intake and sex, by adding an interaction term to model B. There were no significant interactions, but since we previously found sex-differences in the associations of SCB intake and body composition [7], all analyses were performed in the total population as well as stratified for child sex.

As there is inconsistency throughout the literature regarding SCB definition, we performed sensitivity analyses using different definitions of SCBs (excluding fruit juices, and including tea with added sugar). New energy-standardized and sex-specific tertiles of intake were constructed for these definitions. Sensitivity analyses were performed in model B stratified for sex, with cardiometabolic risk factor score as outcome.

To check if certain cardiometabolic components were driving the results for the cardiometabolic risk factor score, analyses were repeated by excluding the components one by one.

To reduce potential bias, missing data on covariates were imputed using the Fully Conditional Specification method (predictive mean matching), assuming no monotone missing pattern [20]. Analyses were performed in each of the 10 imputed data sets separately, and final results were pooled.

A p-value below 0.05 was considered statistically significant. Statistical analyses were performed using SPSS version 21.0.

## Results

### Population

Table 1 shows the characteristics of the 2,045 children and their mothers. Total energy intake was 1314 kcal/day in boys and 1224 kcal/day in girls. Mean (absolute) SCB intake was 7.1 servings per week in boys and 7.0 servings per week in girls. Mothers were on average 32 years old at enrollment in the study, most of them had higher education, and most mothers started folic acid supplementation periconceptionally.

**Table 1:** Characteristics of the children and mothers (n = 2,045)

	Boys (*n* = 1,009)	Girls (*n* = 1,036)
Child characteristics		
Age at FFQ (months)	12.8 (12.2 – 19.4)	12.9 (12.2 – 19.1)
SCB consumption (servings per week)	7.1 (0 – 26.9)	7.0 (0 – 26.0)
Dietary energy intake (kcal/day)	1314 (755 – 2098)	1224 (720 – 2037)
Diet quality score	4.0 (1.9 – 6.4)	3.8 (1.6 – 6.4)
Breastfed (months)	3.5 (0 – 12)	3.5 (0 – 12)
TV watching at age 2 years		
< 1 h/day	50.1 % (505)	52.9 % (548)
> 1 h /day	49.9 % (504)	47.1 % (488)
Age at 6 years visit (years)	5.9 (5.6 – 6.5)	5.9 (5.6 – 6.6)
Weight (kg)	22 (17.6 – 28.4)	21.8 (17.4 – 29.4)
Height (cm)	118.5 (109.9 – 128.6)	117.8 (108.4 – 128.5)
BMI (kg/m^2^)	15.7 (13.7 – 18.7)	15.7 (13.6 – 19.2)
Systolic blood pressure (mmHg)	100 (88 – 118)	101 (88 – 121)
Diastolic blood pressure (mmHg)	59 (47 – 72)	61 (48 – 74)
Pulse wave velocity (m/s)	5.4 (4.0 – 7.6)	5.4 (4.3 – 7.3)
HDL-cholesterol (mmol/l)	1.31 (0.81 – 2.06)	1.28 (0.81 – 2.01)
Triglycerides (mmol/l)	0.98 (0.38 – 2.32)	0.99 (0.43 – 2.42)
Insulin (pmol/l)	116 (17 – 376)	114 (18 – 429)
Preperitoneal fat (cm^2^)	0.34 (0.17 – 0.73)	0.43 (0.20 – 0.95)
Maternal characteristics		
Maternal age (years)	32.3 (22.8 – 40.0)	32.2 (22.7 – 39.7)
Maternal BMI at enrollment (kg/m^2^)	23.2 (18.3 – 34.6)	23.4 (19.1 – 35.2)
Educational level		
Lower	33.2 % (331)	34.4 % (353)
Higher	66.8 % (665)	65.6 % (674)
Folic acid use		
Never	7.4 % (56)	7.0 % (55)
Periconceptionally	63.1 % (475)	63.1 % (231)
Started in first 10 weeks	29.5 % (222)	29.4 % (501)
Smoking during pregnancy		
Never smoked or quit when pregnancy was known	88.3 % (802)	90.8 % (862)
Continued during pregnancy	11.7 % (106)	9.2 % (87)

Values are valid percentages (absolute numbers) or medians (95 % range)

Data on television watching was missing in 55 boys and 66 girls

Data on maternal BMI was missing in 90 boys and 88 girls

### Associations of SCB intake with the cardiometabolic risk factor score

Table 2 shows the associations of SCB intake at 13 months of age, with the cardiometabolic risk factor score at age 6, in the total population and by sex.

**Table 2:** Association of sugar-containing beverage intake with the cardiometabolic risk factor score

	Cardiometabolic risk factor score	
	Total population (*n* = 1,316)	
	Model A	Model B
Low tertile *n = 443*	*Reference*	*Reference*
Medium tertile *n = 443*	−0.00 (−0.13; 0.12)	−0.02 (−0.14; 0.11)
High tertile *n = 440*	0.16 (0.04; 0.28)*	0.13 (0.01; 0.25)*
*Trend*	*p = 0.01*	*p = 0.04*
	Boys (*n* = 681)	
Low tertile *n = 223*	*Reference*	*Reference*
Medium tertile *n = 234*	0.02 (−0.15; 0.19)	0.03 (−0.13; 0.20)
High tertile *n = 224*	0.18 (0.02; 0.35)*	0.18 (0.01; 0.34)*
*Trend*	*p = 0.03*	*p = 0.04*
	Girls (*n* = 635)	
Low tertile *n = 210*	*Reference*	*Reference*
Medium tertile *n = 209*	−0.03 (−0.21; 0.15)	−0.09 (−0.27; 0.10)
High tertile *n = 216*	0.11 (−0.06; 0.30)	0.06 (−0.12; 0.24)
*Trend*	*p = 0.20*	*p = 0.47*

Values are linear regression coefficients (95 % confidence interval) and reflect the difference in outcome (SD scores) for medium and high sugar-containing beverage intake, as compared to the lowest category of intake

Trend tests were performed using tertiles of sugar-containing beverage intake as continuous variable in the model

Model A is adjusted for age at measurements and total energy intake (and child sex in the analysis of the total population)

Model B is additionally adjusted for maternal age, BMI, education level, smoking during pregnancy, folic acid supplement use during pregnancy, breastfeeding of the child, diet quality score, and hours of TV watching at age 2)

*p <0.05

In the total population, the highest tertile of SCB intake was associated with a 0.13SD higher cardiometabolic risk factor score at the age of 6 years (95 % CI 0.01; 0.25), as compared to the lowest quartile, after adjustment for sociodemographic and lifestyle factors.

After stratification for child sex, we observed that boys in the highest tertile of SCB intake had a higher cardiometabolic risk factor score (0.18 SD (95 % CI 0.01; 0.34)), as compared to boys in the lowest tertile of SCB intake. In girls, there was no significant association between SCB at 13 months of age and the cardiometabolic risk factor score (0.06 SD (95 % CI −0.12; 0.24), highest vs. lowest tertile).

Additional adjustment for child height did not materially change these results (data not shown).

### Associations of SCB intake with individual risk factors

Table 3 shows the associations between SCB intake at 13 months and systolic and diastolic blood pressure and pulse wave velocity at age 6, in the total population and stratified by child sex.

**Table 3:** Association of sugar-containing beverage intake with cardiovascular outcomes, in the total population and stratified by sex

	Systolic blood pressure (*n* = 1,950)		Diastolic blood pressure (*n* = 1,950)		Pulse wave velocity (*n* = 1,704)	
	Model A	Model B	Model A	Model B	Model A	Model B
	Total population					
Low tertile *n = 647/578*	*Reference*	*Reference*	*Reference*	*Reference*	*Reference*	*Reference*
Medium tertile *n = 653/559*	0.03 (−0.08; 0.14)	0.02 (−0.09; 0.12)	0.06 (−0.04; 0.17)	0.05 (−0.05; 0.16)	0.00 (−0.11; 0.12)	0.01 (−0.11; 0.12)
High tertile *n = 650/567*	0.05 (−0.06; 0.16)	0.02 (−0.08; 0.13)	0.11 (0.01; 0.22)*	0.09 (−0.02; 0.19)	−0.01 (−0.13; 0.10)	−0.01 (−0.13; 0.11)
*Trend*	*p = 0.34*	*p = 0.67*	***p = 0.03***	*p = 0.10*	*p = 0.81*	*p = 0.86*
	Boys					
Low tertile *n = 317/277*	*Reference*	*Reference*	*Reference*	*Reference*	*Reference*	*Reference*
Medium tertile *n = 320/274*	−0.01 (−0.15; 0.14)	−0.01 (−0.16; 0.13)	0.01 (−0.14; 0.16)	0.01 (−0.14; 0.16)	0.10 (−0.08; 0.28)	0.10 (−0.08; 0.28)
High tertile *n = 317/275*	0.10 (−0.05; 0.24)	0.08 (−0.06; 0.23)	0.14 (−0.00; 0.29)	0.13 (−0.02; 0.28)	0.03 (−0.15; 0.20)	0.03 (−0.15; 0.21)
*Trend*	*p = 0.40*	*p = 0.28*	*p = 0.05*	*p = 0.08*	*p = 0.77*	*p = 0.76*
	Girls					
Low tertile *n = 330/301*	*Reference*	*Reference*	*Reference*	*Reference*	*Reference*	*Reference*
Medium tertile *n = 333/285*	0.07 (−0.09; 0.23)	0.04 (−0.12; 0.20)	0.10 (−0.04; 0.25)	0.08 (−0.06; 0.23)	−0.06 (−0.21; 0.09)	−0.07 (−0.22; 0.08)
High tertile *n = 333/292*	0.00 (−0.16; 0.16)	−0.03 (−0.19; 0.13)	0.08 (−0.07; 0.22)	0.04 (−0.10; 0.18)	−0.05 (−0.20; 0.10)	−0.04 (−0.19; 0.11)
*Trend*	*p = 0.99*	*p = 0.66*	*p = 0.31*	*p = 0.59*	*p = 0.54*	*p = 0.60*

Values are linear regression coefficients (95 % confidence interval) and reflect the difference in outcome (SD scores) for medium and high sugar-containing beverage intake, as compared to the lowest category of intake

Trend tests were performed using tertiles of sugar-containing beverage intake as continuous variable in the model

Model A is adjusted for age at measurements and total energy intake (and child sex in the analysis of the total population)

Model B is additionally adjusted for maternal age, BMI, education level, smoking during pregnancy, folic acid supplement use during pregnancy, breastfeeding of the child, diet quality score, and hours of TV watching at age 2)

*p < 0.05

N per tertile are number of children in the tertiles for the blood pressure measures and for pulse wave velocity, respectively

We found no significant associations with any of the individual risk factors after adjustment in the total population, or stratified by sex. However, for boys, the associations for systolic and diastolic blood pressure were in the direction of a higher cardiometabolic risk factor score. There were no significant associations of SCB intake with mean arterial pressure (data not shown).

Table 4 shows the association of SCB intake at 13 months with metabolic outcomes at age 6. There were no significant associations, but SD scores with children with higher SCB intake tended towards higher cardiometabolic risk, in particular for blood lipids (0.12 SD higher triglycerides (95 % CI −0.01; 0.25) and 0.12 SD lower HDL-cholesterol (95 % CI −0.25; 0.01) for highest vs lowest tertile of SCB intake). Stratified by sex, we observed similar associations, which were strongest in boys. There were no significant associations of SCB intake with total cholesterol, LDL cholesterol and C-peptide levels (data not shown).

**Table 4:** Association of sugar-containing beverage intake with metabolic outcomes, in the total population and stratified by sex

	HDL cholesterol (*n* = 1,390)		Triglycerides (*n* = 1,387)		Insulin (*n* = 1,383)		Preperitoneal fat (*n* = 1,639)	
	Model A	Model B	Model A	Model B	Model A	Model B	Model A	Model B
	Total population							
Low tertile *n = 456/456 456/534*	*Reference*	*Reference*	*Reference*	*Reference*	*Reference*	*Reference*	*Reference*	*Reference*
Medium tertile *n = 466/465 464/554*	−0.06 (−0.19; 0.07)	−0.07 (−0.20; 0.06)	−0.04 (−0.17; 0.09)	−0.04 (−0.17; 0.10)	0.02 (−0.11; 0.14)	0.02 (−0.11; 0.15)	−0.04 (−0.13; 0.06)	−0.05 (−0.15; 0.05)
High tertile *n = 468/466 463/551*	−0.11 (−0.24; 0.02)	−0.12 (−0.25; 0.01)	0.12 (−0.01; 0.25)	0.12 (−0.01; 0.25)	0.02 (−0.11; 0.15)	0.03 (−0.10; 0.16)	0.05 (−0.05; 0.15)	0.02 (−0.08; 0.12)
*Trend*	*p = 0.08*	*p = 0.06*	*p = 0.06*	*p = 0.06*	*p = 0.76*	*p = 0.66*	*p = 0.30*	*p = 0.63*
	Boys							
Low tertile *n = 236/236 2335/253*	*Reference*	*Reference*	*Reference*	*Reference*	*Reference*	*Reference*	*Reference*	*Reference*
Medium tertile *n = 243/243 243/279*	−0.12 (−0.30; 0.06)	−0.13 (−0.31; 0.05)	−0.03 (−0.21; 0.15)	−0.03 (−0.21; 0.16)	0.05 (−0.12; 0.22)	0.07 (−0.10; 0.24)	−0.05 (−0.18; 0.08)	−0.06 (−0.19; 0.07)
High tertile *n = 239/239 237/282*	−0.13 (−0.31; 0.05)	−0.14 (−0.32; 0.04)	0.16 (−0.03; 0.34)	0.15 (−0.03; 0.34)	0.06 (−0.11; 0.22)	0.08 (−0.09; 0.25)	−0.04 (−0.16; 0.09)	−0.06 (−0.19; 0.08)
*Trend*	*p = 0.15*	*p = 0.13*	*p = 0.09*	*p = 0.11*	*p = 0.52*	*p = 0.33*	*p = 0.61*	*p = 0.41*
	Girls							
Low tertile *n = 220/220 221/281*	*Reference*	*Reference*	*Reference*	*Reference*	*Reference*	*Reference*	*Reference*	*Reference*
Medium tertile *n = 222/222 221/275*	0.03 (−0.16; 0.21)	0.02 (−0.17; 0.21)	−0.06 (−0.24; 0.12)	−0.05 (−0.24; 0.13)	−0.02 (−0.21; 0.18)	−0.04 (−0.24; 0.16)	−0.02 (−0.18; 0.09)	−0.05 (−0.18; 0.09)
High tertile *n = 229/228 226/269*	−0.08 (−0.26; 0.10)	−0.09 (−0.28; 0.10)	0.08 (−0.11; 0.26)	0.09 (−0.10; 0.27)	−0.02 (−0.21; 0.17)	−0.03 (−0.23; 0.16)	0.10 (−0.04; 0.24)	0.10 (−0.04; 0.24)
*Trend*	*p = 0.39*	*p = 0.33*	*p = 0.40*	*p = 0.34*	*p = 0.85*	*p = 0.75*	*p = 0.06*	*p = 0.15*

Values are linear regression coefficients (95 % confidence interval) and reflect the difference in outcome (specific SD scores) for medium and high sugar-containing beverage intake, as compared to the lowest category of intake

Trend tests were performed using tertiles of sugar-containing beverage intake as continuous variable in the model

Insulin was root-transformed before standardization

Model A is adjusted for age at measurements and total energy intake (and child sex in the analysis of the total population)

Model B is additionally adjusted for maternal age, BMI, education level, smoking during pregnancy, folic acid supplement use during pregnancy, breastfeeding of the child, diet quality score, and hours of TV watching at age 2)

N per tertile are number of children in the tertiles for HDL-cholesterol, triglycerides, insulin and preperitoneal fat, respectively

### Sensitivity analyses

We additionally performed analysis with the cardiometabolic risk factor score by excluding each component one by one. The association of highest tertile of intake with higher cardiometabolic risk factor score in boys (0.18 SD (95 % CI 0.01; 0.34)), was not clearly driven by a single component.

The association attenuated mostly after excluding triglycerides (0.14 SD (95 % CI −0.02; 0.29)) or HDL-cholesterol (0.14 SD (95 % CI −0.02; 0.29)), and hardly after excluding blood pressure (0.16 SD (95 % CI −0.05; 0.32)) or insulin (0.17 SD (95 % CI 0.00; 0.33)). After excluding body fat percentage, the association became slightly stronger (0.21 SD (95 % CI 0.03; 0.39)).

Sensitivity analyses with different definitions of SCBs showed that the associations remained similar after including tea with added sugar (0.18 SD (95 % CI-0.02; 0.35)), but largely attenuated after excluding fruit juices (0.06 SD (95 % CI −0.10; 0.22)).

## Discussion

In this population-based prospective cohort study, we observed that higher SCB intake at 13 months of age was associated with a higher cardiometabolic risk factor score at 6 years of age. We only observed this association in boys and not in girls, although there was no significant statistical interaction. There were no clear associations of SCB intake with any of the individual cardiovascular or metabolic risk factors.

Our finding that higher SCB intake was associated with a higher cardiometabolic risk factor score in children is in line with studies in adults, which reported a higher risk of metabolic syndrome and type 2 diabetes mellitus [2]. However, the observation that additional adjustment for child weight did not affect the associations for metabolic and cardiovascular risk factors is not in line with a study in adults, which suggested that approximately half of the effect of SCBs on type 2 diabetes was mediated through obesity [21]. This study was performed in women only, and in our population, we found no association of SCB intake with cardiometabolic health in girls, despite the fact that they had an increased BMI in our previous study [7]. In contrast, we previously found that boys with a high intake of SCBs [7] did not have a higher BMI, but we observed in the current study a significant association towards a higher cardiometabolic risk factor score. Thus, our results suggest that SCBs can affect cardiometabolic health directly, and not solely through increasing body weight.

Despite the large amount of research interest on the effects of SCBs on cardiometabolic health [3, 22], there are no studies that assessed the effects of SCB intake below the age of 2 years. One study examined the relation between SCBs and cardiometabolic health in preschool children [5], and this small cross-sectional study observed among 467 children 3–5 years of age, that higher SCB intake was associated with higher LDL cholesterol. Interestingly, the association of SCB intake with BMI and waist circumference in this previous study was not present in these children aged 3–5 years, but was found only in children aged 9–11 years [5]. This gives additional indications that SCB intake may affect metabolic outcomes, without affecting body weight. Unfortunately, this study did not report results stratified by sex in the age-group 3–5 years, and to our knowledge, there are no other studies performed on the relation between SCBs on cardiometabolic outcomes in preschool children. The lack of research on this topic is surprising, as not only dietary patterns track into adulthood [23], but also cardiometabolic risk factors track into adulthood [24, 25]. Thus, early childhood provides an important window of opportunity for prevention.

Previous studies on the associations of obesity with blood lipids and insulin resistance [26, 27] have also found sex differences, which may be related to difference in health behaviors [26]. We previously speculated that the observed differences between boys and girls in the relation of SCBs with BMI might be caused by a lower level of satiety in girls as compared to boys, which was observed in experimental studies in adults [28, 29]. Previous studies also have shown sex differences in development of components of metabolic syndrome and age at onset [30–32], and there might be biological differences, already in children [27]. However, it has been proposed that the vast majority of sex differences in health outcomes are due to social and cultural differences, rather than biological differences [33]. For example, a review on different aspects of parenting in relation to child overweight showed differences in parenting depending on child sex [34]. If a sex interaction truly exists, either biological or social, this could be of importance for future public health programs but further studies are need to elucidate any sex-specific effects. A limitation of our study is that we have only measured cardiometabolic health at one time point. Future studies with repeated measurements of cardiometabolic health care important to clarify any sex differences in the long run.

In sensitivity analyses, we observed that the results attenuated after the exclusion of fruit juices, which suggest that fruit juices contribute to a large extent to the observed associations. This might be related to the fructose in fruit juices, although fructose and glucose do not differ calorically, they may differ in their metabolism [35, 36]. Experimental research in adults even suggested that fructose-sweetened beverages may have more detrimental effects on metabolic outcomes as compared to glucose-sweetened beverages [37]. A large observational study (*n* = 71,346), also done in adults, indeed showed that consumption of fruit juices was associated with risk of type 2 diabetes [38], but it has also been suggested that only fruit juices with added sugar increase the risk of type 2 diabetes mellitus, and 100 % fruit juices do not [39]. To our knowledge this has not yet been studied in children. Our FFQ did not separate 100 % fruit juices from fruit juices with added sugar. Hence, we could not study the role of these different types of fruit juices.

In addition, some other methodological considerations need to be considered in order to appreciate these results. As in any prospective cohort study, loss-to-follow-up has occurred, which might have led to attrition bias. Mothers of children not in the analyses had on average a lower education level, more often did not use folic acid supplements and more often continued smoking during pregnancy, as compared to mothers of children in the analysis. However, they less often continued alcohol use during pregnancy. Hence, there might have been a selection towards a more health-conscious population which may affect the generalizability of our results.

Also, error in estimation of SCB intake might be present. We adjusted SCB intake for total energy to reduce the magnitude of potential systematic measurement error [14], and our FFQ showed good validation for SCB intake against 24 h recalls (intraclass correlation coefficient of 0.76) [7]. Nevertheless, random error might still be present which may have led to an underestimation of the true effect [40].

We performed detailed measures of cardiometabolic health, and combined the individual risk factors into a continuous cardiometabolic risk factor score. The benefits of a continuous score as compared to dichotomous metabolic syndrome definition are that the continuous score is less prone to errors and more sensitive to pick up differences, since more information is being used [17].

However, as this is an observational study, residual confounding might be present. We have selected many potential confounders and observed little change in effect estimates from many socio-demographic and lifestyle variables from children and their parents. Nevertheless, residual confounding might also occur because of poorly measured confounders. For example, we used TV watching as a proxy for sedentary behavior and participation in sports as proxy for physical activity, which were both parent-reported. Because of the lack of a valid measurement of energy expenditure, the activity level of the child might not have been fully covered. Thus, although our results were not influenced by adjustment for TV watching and sedentary behavior, if children with lower energy expenditure have a higher SCB intake, the residual confounding by energy expenditure may have led to an overestimation of our observed associations.

Although causality cannot be established because this study had an observational study design, the available evidence from both observational and experimental studies suggests that SCBs can affect health outcomes of both children and adults unfavorably, and initiatives targeting SCB intake reduction should be explored [41]. We only had information on SCB intake at 1 years of age measured by FFQ. Since it is likely that SCB consumption increases during childhood and an FFQ is better suited for ranking individuals according to their intake than assessing absolute values, we only used ranking of SCB intake by tertiles, rather than absolute amounts. However, even though it has been suggested that diet can track later in childhood [23], we do not know if this also applies to these very young children and if this applies to SCB intake specifically. Future studies with the follow-up measurements from the children at an older age may be helpful in this matter. Nevertheless, our finding of an association between SCB intake at 1 year of age and cardiometabolic health in later childhood indicates that prevention strategies should already start at a very young age.

## Conclusion

In conclusion, we observed that that higher intake of SCBs at the age of 1 year was associated with a higher cardiometabolic risk factor score in school-aged boys. We extend the current literature by showing for the first time that an association between SCBs and cardiometabolic health already exists for consumption of SCBs before the age of 2 years. Future public health policies should target a reduction in SCB intake also in these very young children.
